# The Allentown Connection—A Tribute for Lew Jae-duk, the “Father of Korean Plastic Surgery”

**DOI:** 10.1055/a-2028-6625

**Published:** 2023-05-29

**Authors:** Geoffrey G. Hallock, Joon Pio Hong

**Affiliations:** 1 Division of Plastic Surgery, St. Luke's Hospital, Sacred Heart Division, Allentown, Pennsylvania; 2 Department of Plastic and Reconstructive Surgery, Asan Medical Center, University of Ulsan College of Medicine, Seoul, Republic of Korea

**Keywords:** Lew Jae-duk, Allentown, Korean plastic surgery

## Abstract

In retrospect, the irony of this story began with the first meeting of these co-authors—in of all places, Coimbatore, India, in 2008, at the 12th International Perforator Flap Course. Here the junior author [hereafter “jp”] demonstrated his unparalleled skills in networking, and soon thereafter journeyed some 11,073 km to Allentown, U.S. to peruse the operating room and clinics of the senior author [sic. ggh] in action. Within 2 years jp orchestrated the presentation of the 14th International Perforator Flap Course, so ggh with great anticipation flew only 6,830 miles to reach Seoul, Korea for his first time. But four years more elapsed before ggh returned again to Korea to be a visiting professor, all the while not quite sure why any Korean would want anything from a country doctor who resided in nowheresville Allentown, Pennsylvania. Yet, an extraordinary fact then was to be unveiled, about which ggh was totally ignorant. The pioneer of plastic surgery in Korea, the first Korean to have completed an accredited plastic surgery fellowship, by coincidence had accomplished all this in … Allentown. The collegial relationship that evolved between these co-authors, who met by chance, indeed had a precedent coincidence! Was this “by chance” alone or predestination? Amazingly, in a way similar, the origin of plastic surgery itself in Korea also had Allentown connections. As a tribute to Lew Jae-duk, this important story must be here told, so let us now retrace his past in Allentown so we can find how the future was to be not so far away.

**Figure:**
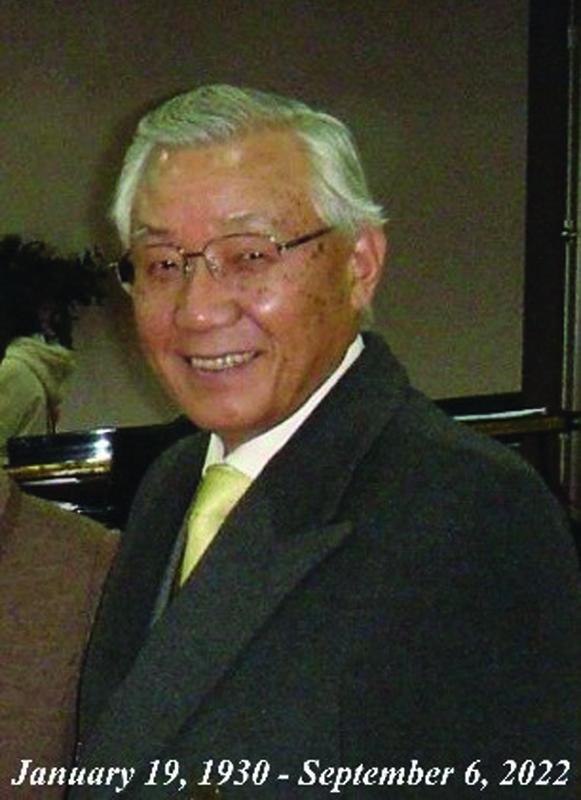
Lew Jae-duk, MD

If indeed the prophecy that “The Past—Mirror of the Future” 1 is true, particularly in these revolutionary times today worldwide, all Homo sapiens must know their basic history to insure the very future of the species itself. Perhaps this may be even more vital for reconstructive surgeons who still wish to be called plastic surgeons? Take the beginning of “plastic surgery” in Korea as our example. Some say the introduction there was by two main groups, “those who were influenced by Dr. Lew Jae-duk and those who were not. 2 ” Our challenge will be to seek the reflections from that mirror of the past that indisputably led to the achievements of that most remarkable man. Lew Jae-duk's inauspicious birth on January 19, 1930, was in Haeju, Whanhae, in what now would be in North Korea ( Fig. 1 ). Those were perilous times. Korea had been first liberated not until he was in third grade. He fled with his father in a snowstorm to South Korea in the winter of 1946. 3 Getting back into school was never difficult for him, first Kyungsung Middle School that became Seoul high school. To move further onward, then a test for applying to the university must be passed; but in those confusing times he was a little late so the only exam option left was at Severance Medical School. Yet, failure was not in his vocabulary, so success in that endeavor changed his youthful goal from becoming a diplomat to now be a doctor. 3 All by chance. So too would be the choice to become a surgeon. Anatomy classes in medical school were found to be interesting, but medical classes were just plain boring. 3 Abruptly all classes stopped, as bombardments everywhere witnessed the eruption of the Korean War. Assuming the role of a civil servant, to escape the frontlines Lew Jae-duk fled southward, only to encounter a gunfight and be hit by a grenade. After 3 to 4 months of dressings, splinting, and recuperation, the now patient hastily returned to Seoul when informed classes at Severance were starting again. Persistence once again led to graduation and a Doctor of Medicine degree from Yonsei Medical College in Seoul in March, 1954. 3 This was followed by an internship that seemed to be just no more than another year as a student in school. So Lew Jae-duk doubted he truly was yet a real doctor. He knew he needed more training. Like so many of his colleagues, his dream was to go to America. His father knew Kim Myunk-sun, who fortuitously at the time was the medical school dean. 3 The dean would recommend him for this course only on the condition that he ultimately “do something that is very rare, and that no one else does. Our country will need it. 3 ” Indeed that would be a prophetic calling.

**Fig. 1:**
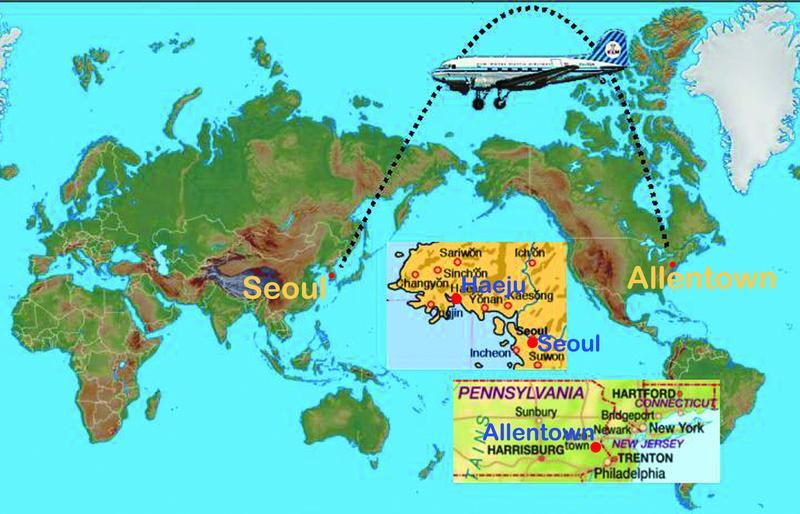
The disparate geographical landmarks in the life of Lew Jae-duk—2 hemispheres apart, i.e., Seoul, Republic of Korea, and Allentown, Pennsylvania, U.S.—yet today united under the umbrella of plastic surgery.

## Arrival in America

Combing the list of teaching hospitals in the Journal of the American Medical Association (AMA), Lew Jae-duk chose and was accepted at St. Mary's Hospital in Hoboken, New Jersey, where he would again be an intern from 1955 to 1956. There communication was painful, every phone call a burden, as he spoke no English at all. To not be left behind, for every hour the other students spent in the library, he had to spend twice as much time. 3 But English he learned. After 6 months, a future specialty had to be chosen. The words “plastic surgery” in the AMA training list caught his attention as a field unquestionably unique. There were only 20 programs available throughout the United States, and he typed all night to apply to all 20. Half did not even respond, and the half that did said they regretted that they were full for a few years. Except for one—who offered an interview. To come to Allentown General Hospital in Pennsylvania, about an hour north of Philadelphia.

And so this is where this story really begins. The customary interview by the chief, a Pennsylvania Dutchman named Kerwin Marcks. Marcks was the first to head what was then known as the Allentown Hospital Department of Plastic and Reconstructive Surgery, from its very startup in 1938. 4 Allentown was and remains a small town nestled within the rolling hills of the Appalachian Mountains, whose existence was to serve the farming communities of the Lehigh Valley. The very first plastic surgery resident only began instruction in 1947, one Allan Trevaskis who actually later was the senior author's (ggh) first partner in 1983. At that time though, Trevaskis was the only plastic surgery resident on duty for every day and for every night, alone for 3 years. 4 Upon finishing this ritual of initiation, he joined Dr. Marcks as an attending in 1950. Together, they established the Allentown Cleft Palate Clinic in 1951, which at the time served the entire state of Pennsylvania, and would be very pertinent to how this story eventually unfolds! Today, this same residency program so started by this pair remains the oldest, non-university-based continuously active Plastic Surgery residency program in the entire United States! 4

**Fig. 2:**
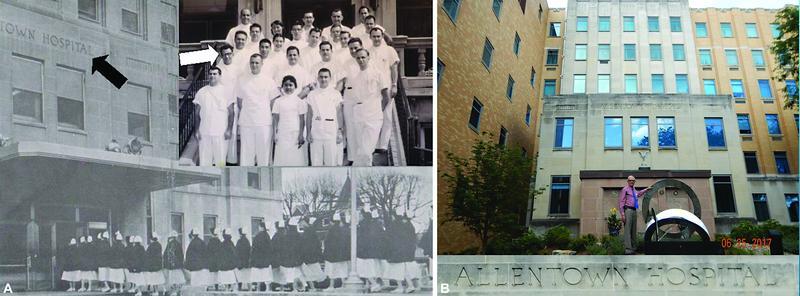
( **A** ) Nurses filing into the main entrance of the Allentown Hospital, as identified by the letters “TOWN“ barely seen visible above them (black arrow), with all the residents including Lew Jae-duk (white arrow) standing on the nearby steps. ( **B** ) This same entrance is now boarded up with ggh seen standing in front, yet the somewhat grimy name “Allentown Hospital” above him on close inspection is better seen as transposed below.

When questioned by Marcks as to why anyone would chose plastic surgery, Lew Jae-duk unhesitatingly replied “I really want to do it because it is a new discipline that I had never heard of in Korea.” 3 And asked “what would be your plans afterwards?” Lew Jae-duk answered that his duty, dignity, and self-esteem would require that he go back to Korea. 3 Impressed, a week later Marcks told him he would be accepted, but only if he promised to pass the plastic surgery board exam. That would require first studying basic fundamentals for 3 years—one year in pathology, then 2 years in general surgery. This would be followed by strictly plastic surgery for 2 more years, for a total of 5 years! Happily leaving New Jersey in 1956, Lew Jae-duk began this residency at the Allentown Hospital, where it is still at the corners of 17th and Chew Streets ( Fig. 2 ).

**Fig. 3:**
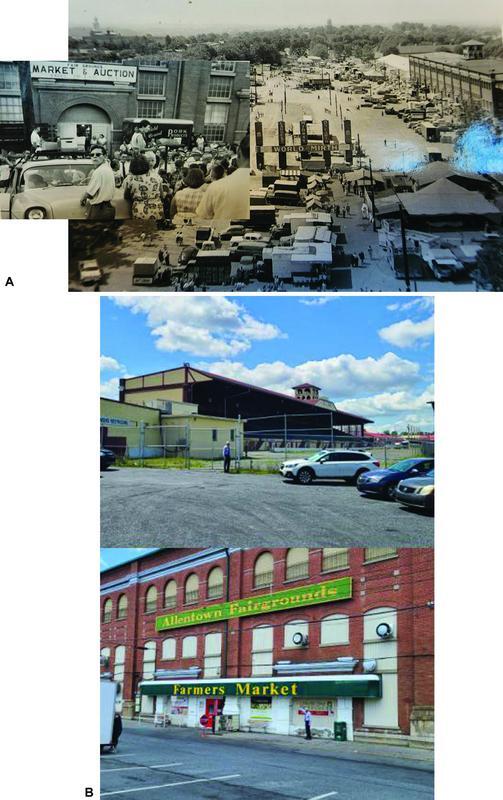
( **A** ) From atop the Allentown Hospital can be seen just across the street the hubbub in the annual county fair next to the Farmer's Market in 1956 that intermingled the farmers' livestock and produce with games of chance for entertainment. ( **B** ) The bleachers looking upon the spacious county fairgrounds (above), and ggh pointing to the same Farmer's Market still sequestered underneath its shelter (below).

The city of Allentown in the 1960 U.S. census had 108,347 inhabitants, and by 2020 had grown to 121,433; a mere speck of dust if contrasted to the 2.36 MILLION in metropolitan Seoul when Dr. Lew had first departed. To realize the apropos scenery of the hospital neighborhood, right across 17th street then and still now is the active Farmer's Market under the Allentown Fairgrounds, typical of the format found in most rural Pennsylvania county seats ( Fig. 3 ).

**Fig. 4:**
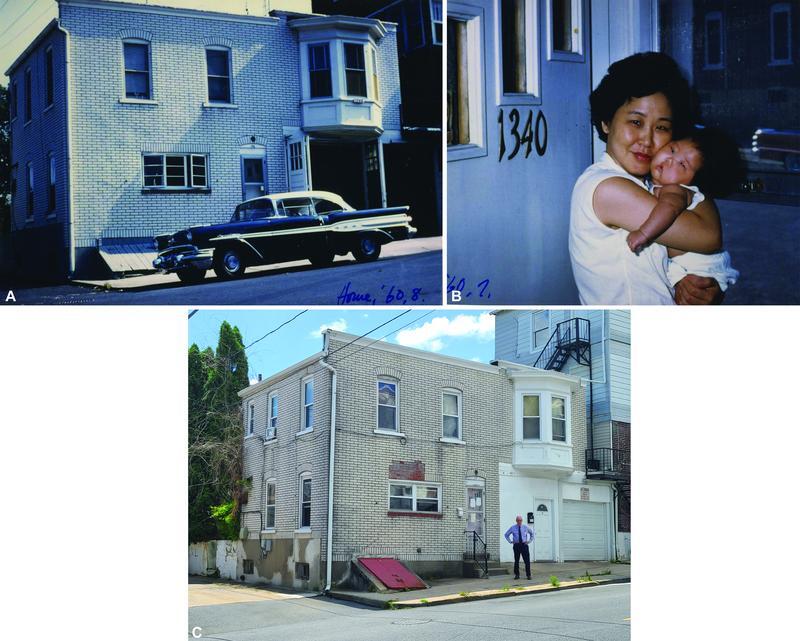
( **A** ) 1340 Union Street, as seen inscribed in the lower right corner “Home, '60, 8” (August, 1960). ( **B** ) The wife of Lew Jae-duk holding their newborn daughter, standing in front of the “1340” door of their home. ( **C** ) 1340 Union Street now with ggh at the front door in 2021. Time here has changed little.

**Fig. 5:**
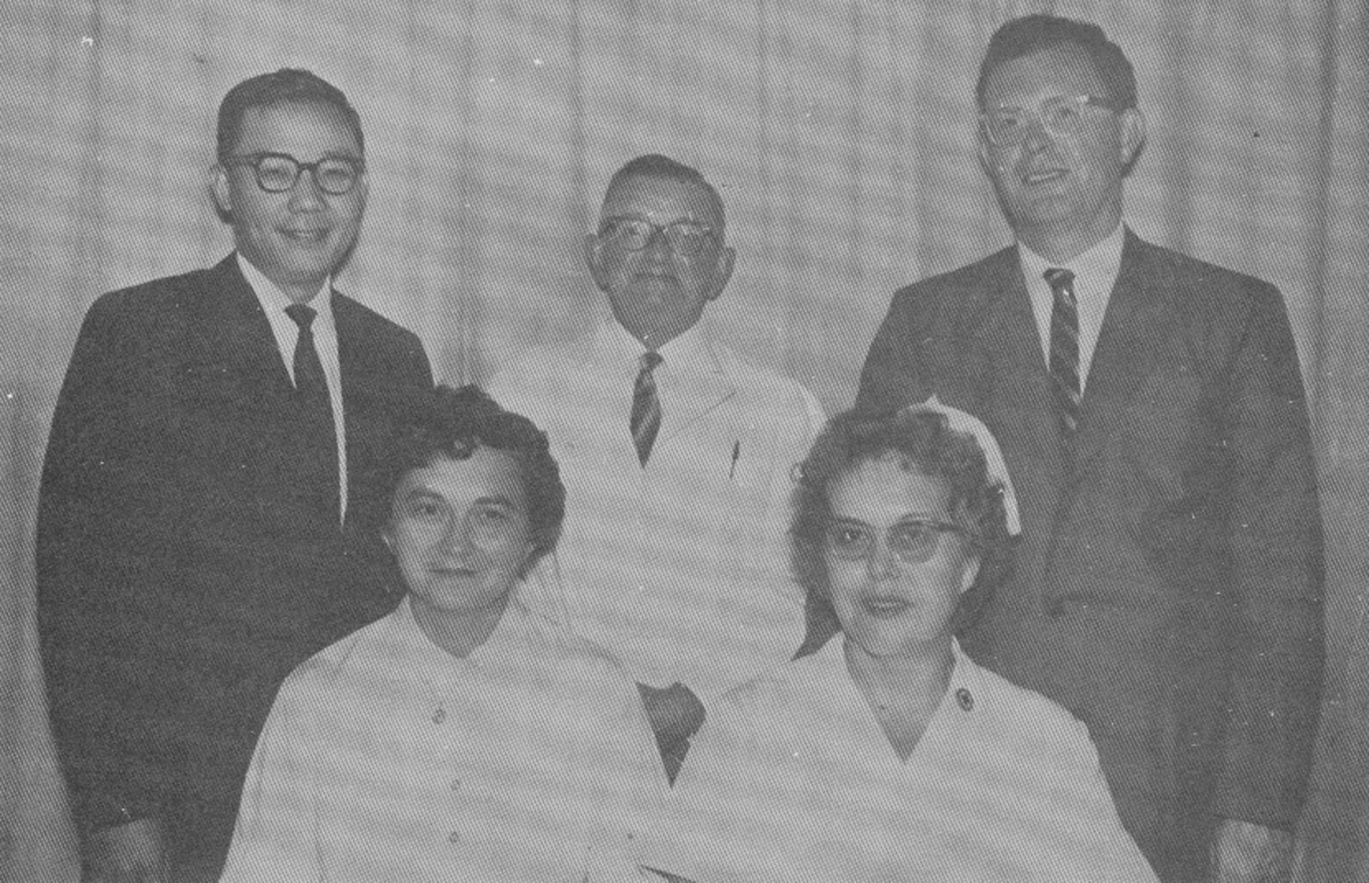
The plastic surgery resident Lew Jae-duk (left), standing next to the chief Kerwin Marcks (center), and his right hand man Allan Trevaskis (right).

Instant availability for emergency call as well as routine duties necessitated nearby living quarters for the new doctor and his wife. That chosen would be found only 8 city blocks away, an easy walking distance to the hospital ( Fig. 4 )! So it finally was to be, at that Allentown General Hospital, a junior resident, Lew Jae-duk met those pioneers of Allentown plastic surgery themselves, Marcks and Trevaskis, who were to shape not just his destiny but that of Korea as well ( Fig. 5 ).

**Fig. 6:**
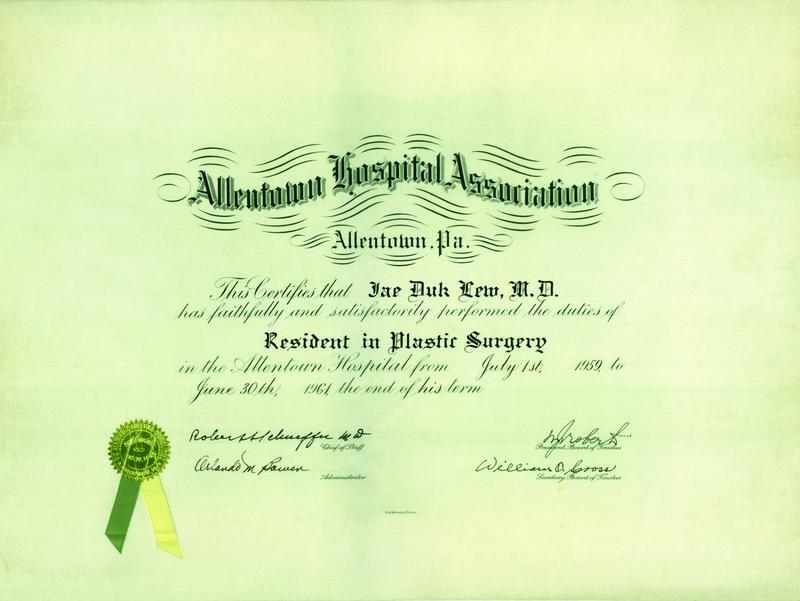
Allentown Hospital Association certificate that Lew Jae-duk had satisfactorily performed his duties as a Resident in Plastic Surgery from 1959 to 1961.

**Fig. 7:**
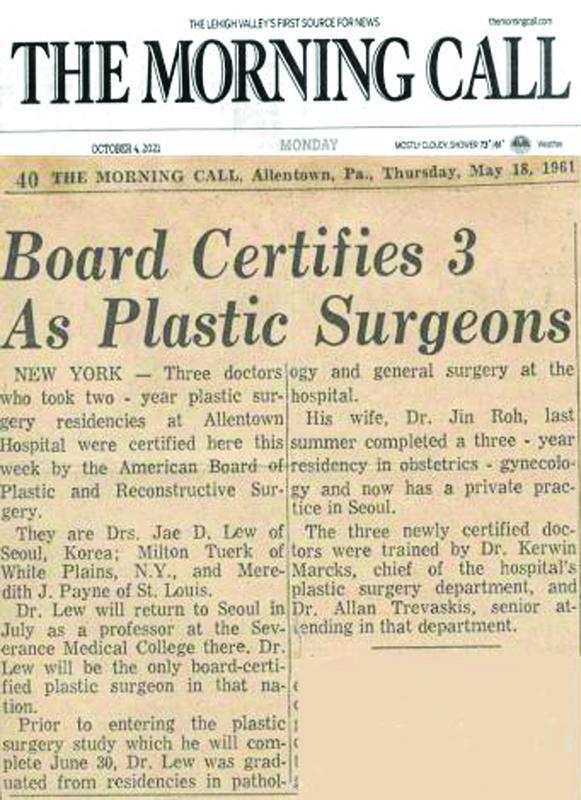
The local Allentown daily paper, THE MORNING CALL, banner heralding the same today (above), and on May 18, 1961, where stating Dr. Lew “will be the only board-certified plastic surgeon in that nation (sic. Korea).”

**Fig. 8:**
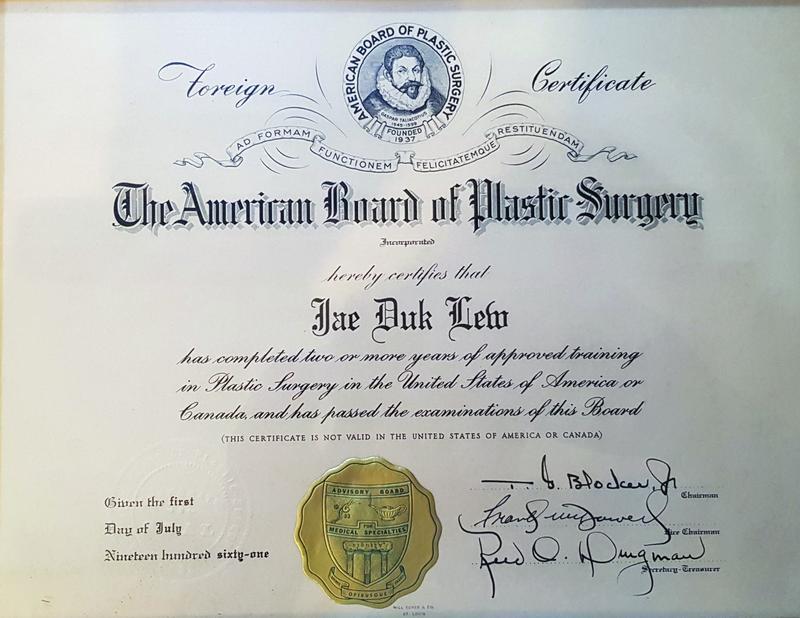
The American Board of Plastic Surgery certified that Lew Jae-duk had completed 2 or more years of approved training in plastic surgery as well as successfully passed the examinations of their board.

Hours and days would be spent in the accident ward on the first floor of the hospital, or treating burns, cleft lips and palates, and flaps as they were, in the operating room on the second floor in room 6, which is still operating room 6 now 60 years later. All went well, as the Allentown Hospital Association certified the successful completion of his term as a resident in Plastic Surgery from July 1, 1959 to June 30, 1961 ( Fig. 6 ). And also so dutifully reported in the local newspaper, The Morning Call ( Fig. 7 ). As he had promised, Lew Jae-duk passed his plastic surgery board exam. In recognition of that achievement, in July of 1961, the American Board of Plastic Surgery provided a certificate documenting completion of his approved training and examination in plastic surgery ( Fig. 8 ). Marcks asked Lew Jae-duk to stay as an associate. But he had more important things to do as also he had promised. He must return to Korea.

## The Return to Korea

**Fig. 9:**
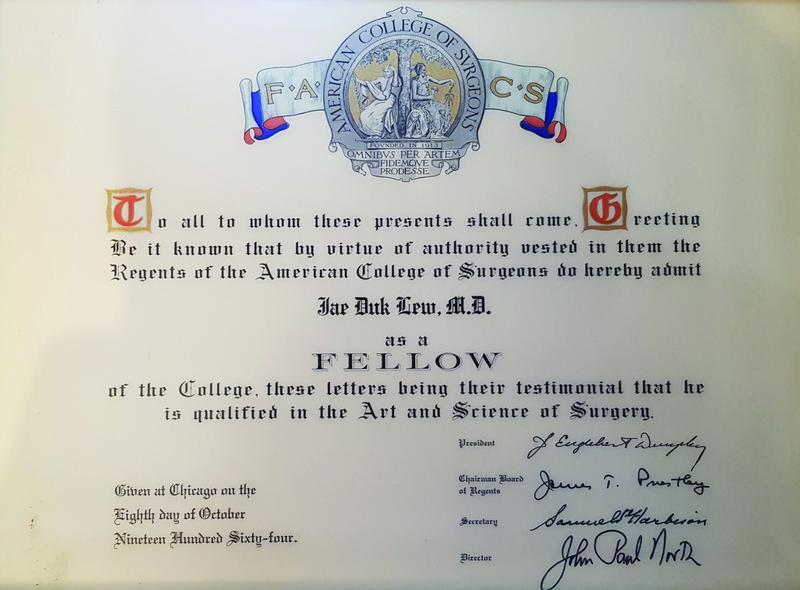
The American College of Surgeons confirmed Lew Jae-duk as a fellow in October, 1964.

On returning to the Republic of Korea, making a plastic surgery department was difficult. The general surgeons controlled surgery, and did not understand why a separate new department was needed. Beginning in August, 1961, as a lowly Instructor, Lew Jae-duk founded the Plastic Surgery Department at Yonsei University in Severance Hospital “thereby establishing plastic surgery as a specialized field of medicine and training specialists in the field for the first time in Korea.” 5 Kim Yong-bae said he thus “was the first to introduce plastic surgery to Korea.” 2 Not to forget his American experience, since there appropriately trained and examined, he became a fellow of the American College of Surgeons, earning the initials F.A.C.S. ( Fig. 9 ).

Rapidly as would be expected and with great effort, he rose up the ranks to eventually become professor and chairman of that department he started (1972–1995). Many of his early 3 6 and even later contributions 7 8 9 10 to the plastic surgery literature scrutinized an amazing volume of cleft lip and palate repairs, where often he preferred a triangular flap technique taught so well by Marcks and Trevaskis. Families in the beginning tried to hide those so afflicted, thinking their child was only sick. Surgery proved to be the easier challenge. Yet, he also reached out to encompass basic science research, 11 12 and sought innovations in flaps which remain so important to every reconstructive practice. 7 13 14 In the early 1970s he obtained a microscope and even did some microvascular experiments in animals, 3 truly an innovator in so many ways.

Lew Jae-duk contributed to the founding of the Korean Society of Plastic and Reconstructive Surgeons (KSPRS) in 1966, 2 and served as their president twice, 1976 to 1978 and 1984 to 1986. Cooperating with the KSPRS, formal resident training programs for plastic surgery were established in 1973, allowing many the same good fortune as had been his in Allentown some 14 years before. Within 15 years of his return, some 20 other medical schools had opened plastic surgery departments in the Republic of Korea. 3 In 1974, the Journal of the Korean Society of Plastic and Reconstructive Surgeons was launched. He subsequently personally contributed numerous articles to what is now known as the Archives of Plastic Surgery, with only some cited here. 15 16 17 18 19 20 21 22

## Epilogue

So must end this brief recapitulation of the trials and tribulations of a true icon of Korean plastic surgery, Lew Jae-duk. ggh will never forget his first journey to Korea as that speaker invited by jp, a guest believing his involvement to be inconsequential, just as was the practice he had left behind in Allentown, Pennsylvania, U.S. On the contrary, many in the audience were shocked by such a coincidence and couldn't wait to disclose to ggh that this was the very same small town that had mentored none other than Lew Jae-duk, the first American trained and board-certified plastic surgeon in all of Korea. How embarrassing ignorance can be, but the truth is what we don't know is what we should. Hopefully, this shortcoming found in this brief story has now been adequately rectified, with the proper respect due shown by so many details about that small Pennsylvania town, that really is not so much different today. None of us must ever forget that Lew Jae-duk rightfully as here been proven “the past” of plastic surgery in Korea, but let there be no doubt he remains forever that mirror shining on all the next generations of plastic surgeons to follow—whether it be in Korea, or Allentown, or for that matter any place in the entire world where a reconstructive surgeon may be found.
